# Case Report: The First Report on *Moraxella canis* Isolation From Corneal Ulcer in a Bulldog

**DOI:** 10.3389/fvets.2022.934081

**Published:** 2022-06-24

**Authors:** Zhihao Wang, Long Guo, Jun Li, Jianji Li, Luying Cui, Junsheng Dong, Heng Wang

**Affiliations:** ^1^College of Veterinary Medicine, Yangzhou University, Jiangsu Co-innovation Center for Prevention and Control of Important Animal Infectious Diseases and Zoonoses, Yangzhou, China; ^2^Joint International Research Laboratory of Agriculture and Agri-Product Safety of the Ministry of Education, Yangzhou, China

**Keywords:** *Moraxella canis*, corneal ulcer, isolation, conjunctival bridge flap, drug resistance

## Abstract

A 5-year-old castrated male bulldog was diagnosed with a corneal ulcer accompanied by edema and conjunctival hyperemia. Ophthalmic examination and microbiological analysis were performed, and the bacteria were found to be gram-negative and globular. The isolated clone was identified as *Moraxella canis* (MZ579539) *via* MALDI-TOF MS and 16S rDNA sequencing. Antimicrobial susceptibility testing showed that the bacteria were sensitive to tetracycline and chloramphenicol, but resistant to levofloxacin and ciprofloxacin. After a conjunctival flap was placed, tobramycin ophthalmic solution and 5% sodium hyaluronate were administered. Following surgery, the ulcer was effectively controlled, and after 3 weeks, the cornea healed. This is the first case report of a canine corneal ulcer associated with *M. canis*, which should be considered when corneal ulceration or keratitis were suspected.

## Introduction

When the epithelium is damaged, a corneal ulcer caused by bacteria is more likely to form. Toxins produced by pathogens or enzymes are released from damaged corneal tissues, which can lead to inflammation and necrosis. As the disease develops, the necrotic tissues shed and ulceration occurs (1). *Pseudomonas aeruginosa, Staphylococcus aureus, Staphylococcus pseud-intermedius*, and *Bacillus cereus* can be involved in canine and feline keratoconjunctivitis and corneal ulcers (2–5). Clinically, bacterial corneal ulcers are often treated with a combination of drugs and surgery. Conjunctival flap surgery is an effective and operationally simple treatment.

*Moraxella canis* (*M. canis*) is a rare, zoonotic, and gram-negative bacterium (6, 7). In 1993, Jannes first proposed that *M. canis* is an *M. catarrhalis*-like strain (8). It has been isolated from various human tissues, such as the lymph node of a patient with alcoholism (9), a foot ulcer of a patient with diabetes (10), and the septic joint cavity of a patient with multiple myelomas (11). The first reported case of keratoconjunctivitis caused by *M. canis* in animals was found in a camel herd in 2010 (12). The pathogenicity of *M. canis* in dogs and cats is still unclear. However, recent studies suggest that *M. canis* is a commensal bacterium present in the oral cavities of both dogs and cats (8). In this study, *M. canis* was isolated from a canine corneal ulcer. Treatment was based on the results of antimicrobial susceptibility testing, and the conjunctival flap surgery was also performed.

## Case Description

**Figure 1:**
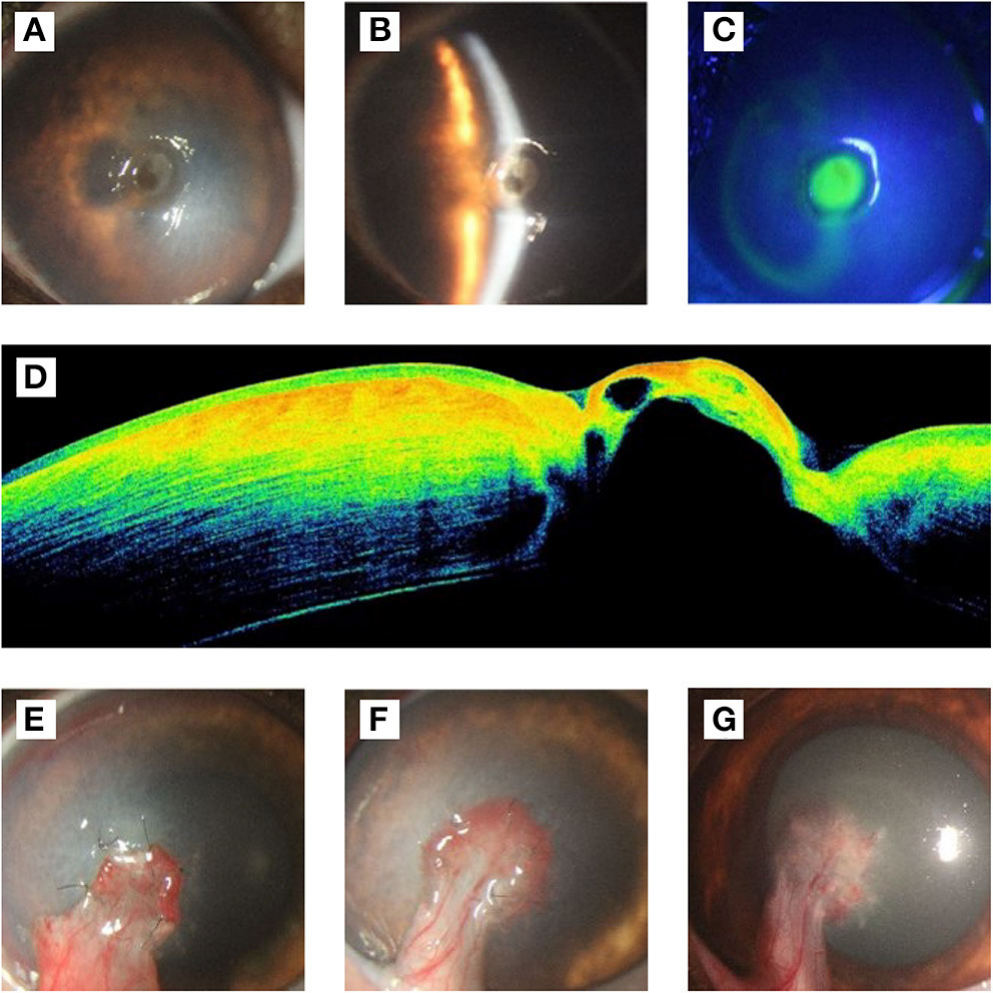
Ophthalmological examination results before and after the operation. **(A)** Frontal view of cornea, there was a circular ulcer (approximately 3 mm in diameter) at the center of the cornea, and the anterior chamber of the left eye was invisible. **(B)** Slit-lamp examination. The result showed that the corneal epithelium was disappear in ulcer, stromal around the ulcer was edema, and the depth of the ulcer was about 70% of the corneal thickness. **(C)** Fluorescein sodium staining showed positive. **(D)** The results of OCT examination showed that the epithelium and endothelium in the ulcer area were missing, part of the stroma and Descemet's membrane bulged, and the reflection of the stroma in the ulcer area was higher, which was suspected to be related to edema. **(E)** On the 7th day after operation, the conjunctival flap grew well and closely with the corneal ulcer area. The corneal edema area enlarged compared with that before operation. **(F)** On the 14th days after operation, the new blood vessels extended from the conjunctival flap to the corneal tissue, and the sutures were removed. The edema subsided gradually compared to 7 days after surgery. **(G)** On the 21st days after operation, the corneal edema subsided completely, and the transparency recovered around the ulcer.

A five-year-old castrated male bulldog, weighing 10.3 kg, presented with photophobia, conjunctival hyperemia, and corneal edema with ulceration in the left eye. The dog had no history of other diseases. There was circular ulceration (approximately 3 mm in diameter) at the center of the cornea, and the anterior chamber of the left eye was invisible (Figure 1A). Slit-lamp biomicroscope (Portable Keeler, YZ3, 66 vision-Tech, China) examination showed stromal edema around the ulcer, and the depth of the ulcer reached 70% of the corneal thickness (Figure 1B). Fluorescein sodium staining was positive, and the positive area was consistent with the size of the ulcer (Figure 1C). Descemetocele was found when preparing for optical coherence tomography (OCT; CIRRUS HD-OCT 400, ZEISS, Germany), which was confirmed by the OCT (Figure 1D). The OCT results also showed that endothelial cells had disintegrated and disappeared, indicating that corneal perforation was possible and could be prevented during surgery.

## Diagnosis and Confirmation Procedure

**Figure 2:**
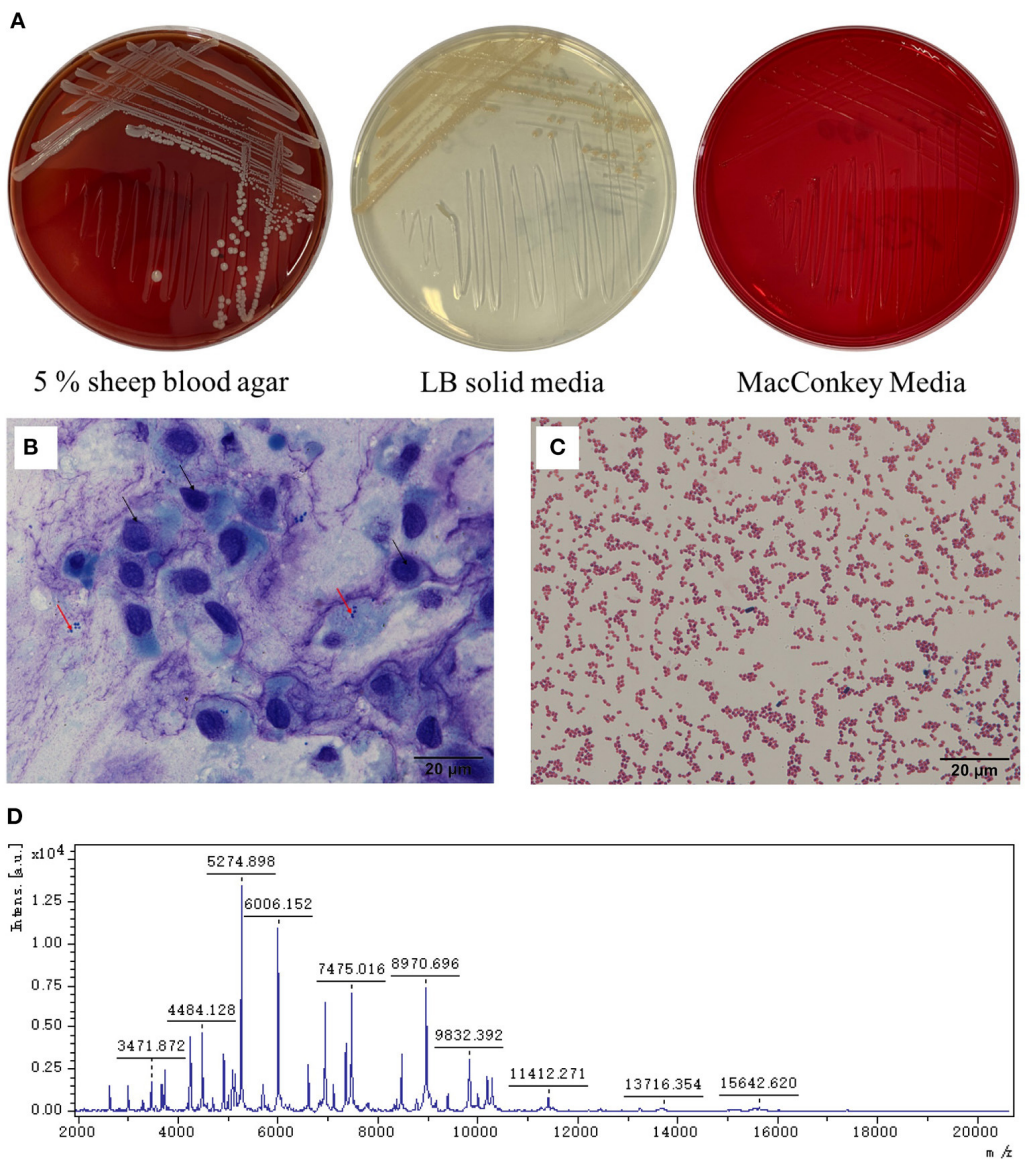
Staining and microscopic examination from the corneal ulceration foci sample collected by sterile cytobrush and pathogen isolation. **(A)** The colonies were white and β-hemolysis in 5% sheep blood agar, light yellow in LB solid medium, and no colonies grown on the MacConkey media. **(B)** The sample was stained by Wright-Giemsa stain. The black arrow refers to the shed corneal cells, and the red arrow refers to the bacteria (10 × 100). **(C)** The bacteria from the culture were dyed by the Gram stain solution and by microscope (10 × 100). **(D)** The mass spectrum peak of the isolated strain.

The affected dog was placed on the examination table and the head was held. The examiner was wearing sterile gloves. The eyelids were pulled back and the eye was flushed with physiological saline solution. Following this, a sterile cytobrush was rolled over the ulceration. The sample was sent to a lab for bacterial isolation and Wright-Giemsa staining. Corneal epithelial cells (black arrow) and bacterial cells (red arrow) were found (Figure 2C). The intraocular pressure (IOP; Icare® TONOVET) of the left eye had decreased by 7 mmHg. The Schirmer tear test value was 28 mm/min, which had increased than the normal range. The menace response was negative and fluorescein staining was positive. Based on slit-lamp examination and Wright-Giemsa staining results, the dog was diagnosed with a bacterial corneal ulcer. Before surgery, the bulldog underwent a complete general physical examination, which was normal, and complete blood counts were within the normal range.

Chloramphenicol eye drops were administered, 3 h before surgery, every 30 min for preoperative preparation. After the operation, chloramphenicol eye drops and 5% sodium hyaluronate were administered hourly for 3 months. Autologous serum was only used within 3 weeks of operation. Seven days post-surgery, the corneal edema area had grown larger than the preoperative area, while the conjunctival graft grew well (Figure 1E). The graft had completely integrated with the cornea and healed well, 14 days post-surgery. Meanwhile, the sutures were removed (Figure 1F). Twenty-one days post-surgery, the corneal edema subsided, and the non-ulcerated area of cornea transparency was deemed recovered (Figure 1G).

Samples of the corneal ulcer were collected using a cytobrush and inoculated on 5% sheep blood, Luria-Bertani (LB), and MacConkey agar plates. These were incubated under aerobic conditions at 37°C for 18–24 h. Based on colonial morphology, single colonies were selected for purification culture. On the agar plates with 5% sheep blood, the bacterial colonies were white, moist, and β-hemolytic, with neat edges. On the LB agar plates, the colonies were yellow-white and moist, with neat edges. No colonies grew on the MacConkey agar plates (Figure 2A). Gram staining showed that the bacteria were gram-negative and globular (Figure 2D). Biochemical testing showed that the isolated strain was positive for oxidase, catalase, nitrate reductase, and tributyrate esterase, and negative for phenylalanine dehydrogenase, urease, and gelatin hydrolase.

The colonies (YZJSC 760) were purified twice, according to the methods in previous reports (13–15). Matrix-assisted laser desorption/ionization time-of-flight mass spectrometry (MALDI-TOF MS; MALDI Biotype, Bruker Daltonics, Bremen, Germany) was then performed. Sterile toothpicks were used to obtain a single colony, which was then smeared onto the MALDI-TOF MS target plate. Using specific fingerprints, the mass spectrum was compared to that of the MBT BDAL library (DB5989_specieslist total) for species identification. Based on the mass spectrum (Figure 2D), the bacterium was identified as *M. canis* and the Biotyper score was 2.030, which is in the “secure genus identification; probable species identification range”.

Next, 16S rDNA sequencing was applied to identify the isolates (16). The amplified products were recovered from the agarose gel using a gel extraction kit (TIANGEN, Nanjing, China), and the purified amplicons were detected by Tsingke Biotechnology Co., Ltd. (Nanjing, China). The sequences of the strain were deposited in GenBank under the accession number LT899982.1. Finally, the sequences were compared with those in the NCBI databases using a BLAST search, which confirmed that the isolated strain was *M. canis*. The sequence data were submitted to GenBank with the accession number MZ579539.

**Figure 3:**
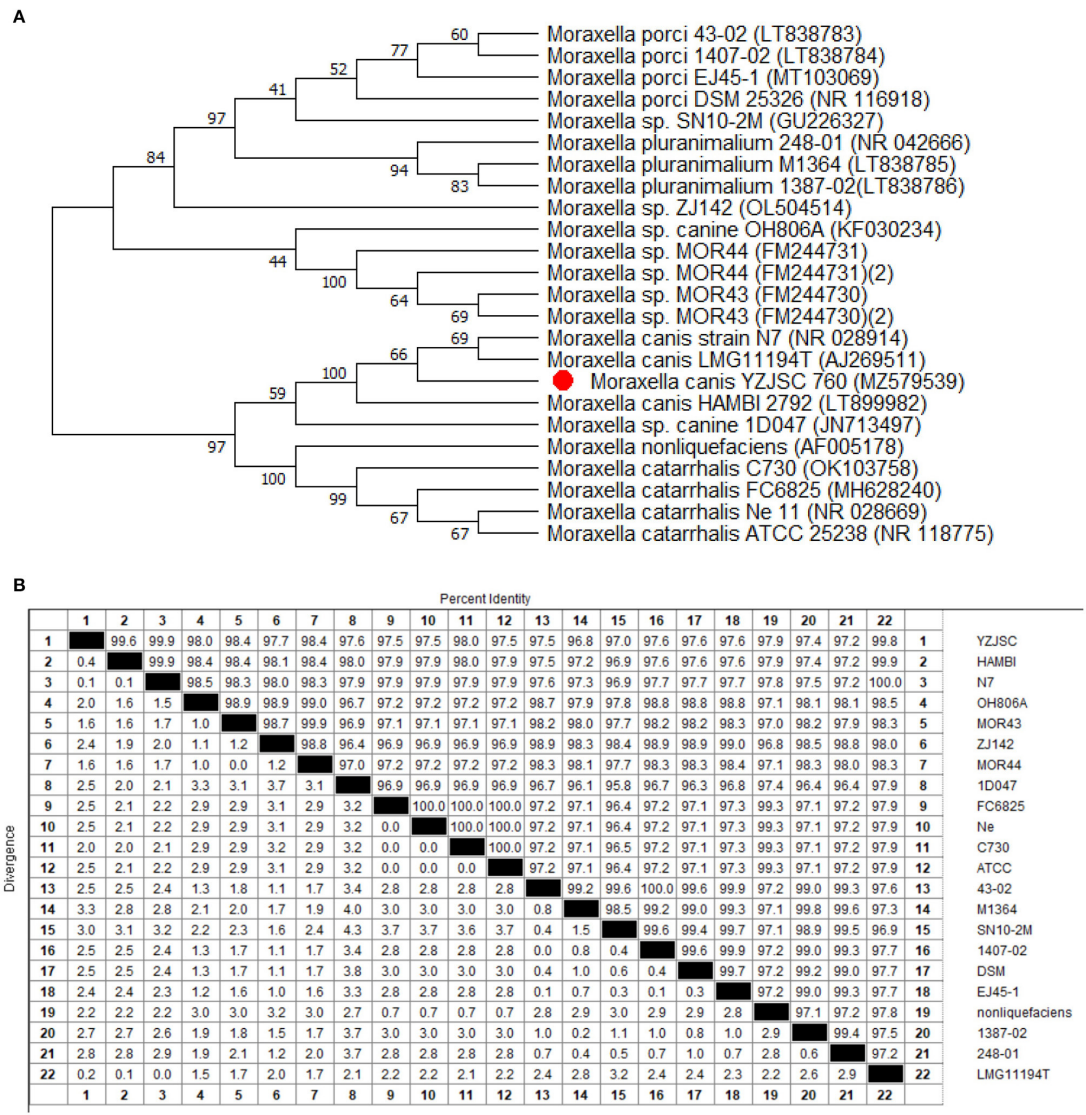
Phylogenetic tree and homology analysis. **(A)** Phylogenetic tree of the genus *Moraxella* based on cluster of 16S rDNA gene sequences by the unweighted-pair group method with averages (UPGMA). Strains are arranged according to the distance between the corresponding sequence and the 16S rDNA gene sequence of *M. canis* AJ269511 and NR 028914. **(B)** Homology analysis showed that the YZJSC strain in this paper was high homology with HAMBI, N7, MOR43, MOR44, and LMG11194T.

Phylogenetic analysis was performed based on the 16S rDNA gene sequence using the neighbor-joining (NJ) method. The NJ tree was constructed using the MEGA11 software, and the robustness of the phylogenetic analysis was determined by bootstrap analysis with 1,000 replicates (17). Figure 3 presents a 16S rDNA gene-based phylogenetic tree comprising most of the genus *Moraxella*, with sequence similarities of 99.8% for strain AJ269511 and 99.9% for strain NR 028914.

Antimicrobial susceptibility testing (AST) for YZJSC 760 was performed using the agar disc diffusion method, according to the guidelines of the Clinical Laboratory Standards Institute (18). Briefly, the bacteria were inoculated into Mueller-Hinton agar supplemented with 5% defibrinated horse blood and 20 mg/L β-NAD, then incubated at 35°C with 5% CO_2_. The inhibition zone diameter was determined after 18 h. Six antimicrobial agents were selected, namely tetracycline (30 μg), chloramphenicol (30 μg), neomycin (30 μg), ciprofloxacin (5 μg), erythromycin (15 μg), and levofloxacin (5 μg). *Haemophilus influenza* strain ATCC 49766 was used for quality control. All antimicrobial agents were purchased from Hangzhou Microbial Reagent, Hangzhou, China. The AST results (Table 1) show that the bacterium was sensitive to tetracycline, chloramphenicol, rifampicin, tobramycin, and neomycin, but resistant to levofloxacin and ciprofloxacin.

**Table 1:** The results of antimicrobial susceptibility test.

**Name**	**Sensitivity judgment/mm**		**Diameter**		**Sensitivity**
	***S*≥**	***R*<**	**IS**	**QC**	
Tetracycline	28	25	30	33	S
Chloramphenicol	30	30	36	35	S
Neomycin	23	20	21	14	I
Ciprofloxacin	26	26	18	37	R
Erythromycin	23	20	21	14	I
Levofloxacin	26	26	0	36	R

*R, Resistant; S, Sensitive; I, Intermediary; Diameter, Inhibition zone diameter (mm); IS, Isolated strains of M. canis; QC, Quality control strain of Haemophilus influenzae ATCC 49766*.

## Discussion

Corneal ulcer in dogs has been reported as an important ophthalmic disorder (19, 20). A survey from 2001 to 2014 in Austria about canine ophthalmic diseases showed that 46 of the 245 dogs with corneal diseases got corneal ulcers (or 18.8%) (21). Several studies have revealed that the incidence of the corneal ulcers was related to breeds and the brachycephalic dogs were susceptible to the disease (22, 23). In 2017, a survey about the breeding information in 2,168 brachycephalic dogs has been conducted, and the results showed that there were 15.4% of dogs had ever suffered from a bacterial corneal ulcer, and nearly one-quarter breed (22.9%) was the Pug (24). The common causes of corneal ulcers in dogs included trauma, foreign bodies, infection, and inadequate lacrimal secretion or others (25, 26). A survey from 2015 to 2016 in Thailand has indicated that 32 ulcers samples, 26 (81.3%) samples yielded culturable microorganisms with 24 bacterial isolates, and 7 fungal isolates (27). Additionally, in the United Kingdom, the survey from 2014 to 2018 indicated that 32.7% of cases were caused by bacteria in 336 dogs with melting corneal ulcers (28). According to the investigations, the major bacteria, which were separated from the canine corneal ulcer, included *Staphylococcus spp*., *Streptococcus spp., Pasteurella multocida, Pseudomonas aeruginosa, Corynebacterium spp., Neisseria spp*., and *Escherichia coli*. (5, 29, 30). Some corneal ulcers can be severe and progress rapidly, resulting in vision loss. Most corneal ulcers are difficult for keepers to notice in the early stages but in this case, as the disease developed, the animal exhibited photophobia and lacrimation, which attracted the keeper's attention, and the animal was presented to the hospital with ophthalmic complaints. At this time, a corneal ulcer was found with large-scale corneal edema. In some cases, corneal perforation or descemetocele formation develops.

In this case, the affected dog, presented with severe corneal ulcer and edema, is complicated by the descemetocele. OCT was used to fully examine the corneal ulcer and endothelium, which provided more information about the risk of corneal perforation and potential surgical complications. A conjunctival flap was used to cover the ulcer and promote corneal recovery. A thin flap of the conjunctiva was transplanted to cover the cornea and provide metabolic and nutritional support. Due to the depth of the corneal lesion, autologous platelet-rich plasma treatment was also administered for 3 weeks (31). After the operation, tobramycin and 5% sodium hyaluronate were administered. The keeper actively cooperated and consented to all veterinary treatment throughout the treatment process.

In this case, the corneal ulcer was infected by *M. canis*, producing severe clinical symptoms associated with a poor prognosis. The clinical features of this case were similar to those of a previous case of corneal ulceration caused by *Pseudomonas aeruginosa* (32). Studies show brachycephalic (short-nosed) dogs have higher morbidity levels associated with bacterial keratitis than other breeds due to their short respiratory tracts and bulging eyeballs (33). This means that keepers of brachycephalic dogs should pay close attention to their eyes, which are susceptible to external irritation.

The pathogen isolated in this study was identified using MALDI-TOF MS and verified by 16S rDNA sequencing. The results were consistent and reliable. Compared with the 16S rDNA identification, MALDI-TOF MS is more accurate, cheaper, and more efficient, which is important to note when selecting appropriate treatment and surgery plan. As seen in the phylogenetic tree, the isolate showed high homology with another *Moraxella*, which was isolated from an ulcerated metastatic lymph node (AJ269511.1 and NR028914). The mid-point rooted phylogenetic tree further confirms that the *M. canis* isolated from this case belongs to the same major clade isolated in Belgium and the USA.

Until recently, the genus *Moraxella* contained approximately 20 species. *M. atlantae, M. lacunata, M. lincolnii, M. nonliquefaciens, M. osloensis*, and *M. phenylpyruvica* are members of the normal microbiome within the human respiratory tract (34). Animal strains include *M. bovis* (isolated from the conjunctival sac and nasal cavity of healthy cattle and other animals, including horses), *M. boevrei* and *M. caprae* (mainly isolated from the nasal cavity of healthy goats), *M. canis* (isolated from the oral mucosa of dogs and cats as well as the conjunctival sac of camels), *M. caviae* (mainly isolated from guinea pigs), *M. cuniculi* (mainly isolated from the mouth of rabbits), *M. ovis* and *M. oblonga* (mainly isolated from the conjunctiva and upper respiratory tract of sheep), and *M. equi* and *M. bovoculi* (isolated from horses with conjunctivitis) (35).

*Moraxella* is associated with various infections, such as conjunctivitis, keratitis, meningitis, septicemia, endocarditis, arthritis, and otolaryngological infections (9, 36, 37). *M. catarrhalis* can cause sinusitis and otitis through contiguous spread from respiratory tract infections (36). *M. lacunata* has been involved in eye infections (31) and infectious endocarditis (38, 39), while *M. bovis* is the primary etiological agent of infectious keratoconjunctivitis, highly contagious disease in cattle (40, 41). *M. ovis* is the critical pathogenic bacteria in ovine infectious keratoconjunctivitis (42), and *M. equi* can cause infectious keratoconjunctivitis in horses (43) and camels, such as the outbreaks first described in 2010 (12). However, there is currently no other evidence that canine corneal ulceration can be induced by *M. canis*.

In this case, the dog was treated using conjunctival flap surgery. Tobramycin was administered to control the infection, and 5% sodium hyaluronate was used to repair the tear film. After 3 weeks, the corneal infection remained, and the corneal edema disappeared. Due to the limited number of cases, a larger epidemiological investigation is still required to verify the relationship between *M. canis* and corneal ulceration. Further studies are recommended into cases of this nature.

## Data Availability Statement

The datasets presented in this study can be found in online repositories. The names of the repository/repositories and accession number(s) can be found below: https://www.ncbi.nlm.nih.gov/genbank/, MZ579539.

## Ethics Statement

The animal study was reviewed and approved by Yang Zhou University (No: 202011003). Written informed consent was obtained from the owners for the participation of their animals in this study.

## Author Contributions

HW and ZW designed the study. LG carried out primary canine treatment. ZW and LG performed patient care and examinations. HW, ZW, and LG oversaw manuscript drafting. ZW, LG, LC, JiL, JD, and HW supervised the study and wrote the manuscript. Each author had agreed with our eventual version of the manuscript. All authors contributed to the article and approved the submitted version.

## Funding

This work was supported financially by 333 High-Level Talent Training Project of Jiangsu Province, China, Jiangsu Postgraduate Research and Innovation Plan (KYCX21_3273), National Key R&D Program (2016YFD0501010), Priority Academic Program Development of Jiangsu Higher Education Institution (PAPD), and Top-notch Academic Programs Project of Jiangsu Higher Education Institutions (TAPP).

## Conflict of Interest

The authors declare that the research was conducted in the absence of any commercial or financial relationships that could be construed as a potential conflict of interest.

## Publisher's Note

All claims expressed in this article are solely those of the authors and do not necessarily represent those of their affiliated organizations, or those of the publisher, the editors and the reviewers. Any product that may be evaluated in this article, or claim that may be made by its manufacturer, is not guaranteed or endorsed by the publisher.
